# Effects of a ferment soy product on the adipocyte area reduction and dyslipidemia control in hypercholesterolemic adult male rats

**DOI:** 10.1186/1476-511X-7-50

**Published:** 2008-12-16

**Authors:** Nadia Carla Cheik, Elizeu Antônio Rossi, Ricardo Luís Fernandes Guerra, Neuli Maria Tenório, Cláudia Maria Oller do Nascimento, Fabiana Pavan Viana, Marla Simone Jovenasso Manzoni, Iracilda Zeponni Carlos, Patrícia Leão da Silva, Regina Célia Vendramini, Ana Raimunda Dâmaso

**Affiliations:** 1 UFU/Federal University of Uberlândia, Uberlândia, Minas Gerais, Brasil; 2 UFSCar/Universidade Federal de São Carlos, São Paulo, Brasil; 3 UNESP/Universidade Estadual de São Paulo, São Paulo, Brasil; 4 UNIFESP/Universidade Federal de São Paulo, Escola Paulista de Medicina, São Paulo, Brasil

## Abstract

**Background:**

Available data on the effects of a fermented soy product enriched with *Enterococcus faecium *and *Lactobacillus Jugurti *on circulating lipids and adiposity are not completely settled. This study aimed to observe the effects of a fermented soy product enriched with *Enterococcus faecium *and *Lactobacillus Jugurti *on central obesity and dyslipidemia control in Wistar adult male rats.

**Methods:**

Over a period of 8 weeks, animals had *"ad libitum" *food intake and water consumption as well as body weight and food consumption was monitored. The animals were assigned to four different experimental groups: Control Group (C); Control + Fermented Product Group (CPF); Hypercholesterolemic diet group (H); and Hypercholesterolemic + Fermented Product Group (HPF). The HPF and CPF groups received an intragastric administration of 1 ml of fermented product daily. After the experimental period the animals were killed by decapitation, blood was collected to measure cholesterol, triglycerides and HDL-cholesterol plasma concentration. Adipocyte circumference, lipolysis and lipogenis rates were measures using epididymal and retroperitoneal white adipose tissues.

**Results:**

The results demonstrated that 1 ml/day/rat of the fermented soy product promoted important benefits such as reduced cholesterolemia in hypercholesterolemic diet group and the adipocyte circumference in both control and hypercholesterolemic diet group.

**Conclusion:**

The fermented soy product enriched with *Enterococcus faecium *and *Lactobacillus Jugurti *decreased circulating lipids levels and reduced adipocyte area in rats.

## Background

The term probiotic refers to live micro-organisms which when administered in adequate amounts confer a health benefit on the host [1]. Probiotic bacteria have been the focus of much scientific and commercial interest due to a range of possible health effects of these bacteria such as on lipid metabolism [2,3]. The most widely studied probiotic bacteria were *Lactobacillus GG*, *Lactobacillus acidophilus*, *Bifidobacterium bifidum *and *Enterococcus faecium *[2-4].

Probiotic dairy products are considered to have functional properties because the probiotic bacteria added to the regular fermentation cultures provide therapeutic benefits such as modification of the immune system, reduction in cholesterol, alleviation from lactose intolerance and faster relief from diarrhea [5].

Available data on the effects of probiotic bacteria on lipid metabolism are controversial. It has been reported that *Lactobacillus acidophilus *[6] or *Streptococcus faecalis *[7] had hypocholesterolemic functions and the cholesterol-lowering mechanism depends heavily on binding of dietary cholesterol by *Lactobacillus acidophilus *or *Streptococcus faecalis*. Zacconi et al. [8] shown that *Enterococcus faecium *had a higher hypocholesterolemic effect in axenic mice than *Lactobacillus acidophilus*. In another study, *L. acidophilus *and a probiotic mixture decreased serum cholesterol concentration of rats fed with fat-and cholesterol-enriched diet while the *Streptococcus faecalis *failed to promote this effect [9]. On the other hand, in mice fed with control diet [10] and in rats fed with hypertriglyceridemic diet [11] the *L. acidophilus *had no effect on plasma cholesterol level. In a normocholesterolemic women and men, fermented milk with a bacteria culture containing *Enterococcus faecium *promoted a rapid reduction of LDL-cholesterol, but during a long-term intake (6 month) the reduction of LDL-cholesterol was similar to the placebo product [12].

Nowadays the soybean is another alimentary source that gets attention from the scientific community. Several studies have been shown that the soy protein, with or without dietary cholesterol, lowers plasma cholesterol and triacylglycerol concentrations in rats [13-15]. In contrast, Moundras et al. [16] reported that when rats were fed soybean protein at a suboptimal level (13%), serum cholesterol concentration was significantly higher than that in rats fed with 13% casein diet, and this higher cholesterol level was counteracted by supplementation of the diet with 0.4% methionine.

Some studies in human subjects have shown a significant hypocholesterolemic effect of soy protein in hypercholesterolemic subjects [17-20]. However, the hypocholesterolemic effect of soy protein also has been shown to be minimal or negligible in normocholesterolemic subjects [21,22]. In addition, it has been reported that the soy protein versus the casein diet can reduce body fat and serum insulin levels in rats [23].

Manzoni et al. [24], reported that the soy product fermented by *Enterococcus faecium *and *Lactobacillus Jugurti *supplemented with isoflavones had beneficial effects on white adipose tissue in juvenile male rats, leads to a decrease in adipocyte size. However, the consequences of *Enterococcus faecium *and soy protein intake on circulating lipids and adiposity area are not completely settled. In this sense, the purpose of the present study was to examine the effect of ferment soy product enriched with *Enterococcus faecium *and *Lactobacillus Jugurti *in rats fed with control diet or hypercholesterolemic diet on circulating lipids levels and adipocyte area.

## Results

### Percentage of Body Weight Gain and Total Food Intake

The results of body weight and food intake are shown in Table 1. The percentage of body weight gain and total food intake were increased in H group as compared to the C and HPF groups. No significant differences in the percentage of body weight and total food intake were observed among C, CPF, and HPF groups.

**Table 1:** Body weight gain (%) and the total food intake (g) in rats fed different diets during 8 weeks.

Group	Body weight gain	Total food intake
C	64.06 ± 4.18	1108 ± 55.04
CPF	73.55 ± 1.99	1193 ± 21.84
H	94.86 ± 3.64^+^	1405 ± 33.64^+^
HPF	77.44 ± 2.26*	1250 ± 34.50*

Values are means ± SEM (n = 8).*p < 0.05 comparing C × CPF and H × HPF; ^+^p < 0.05 comparing C × H. Control Group (**C**); Control + Fermented Product Group (**CPF**); Hypercholesterolemic Diet Group (**H**); and Hypercholesterolemic + Fermented Product Group (**HPF**).

### Retroperitoneal and Epididymal adipose tissues

Table 2 shows that there were no significant differences between C and CPF groups when RET (absolute and relative) and EPI (relative) weight as compared. In the HPF group the RET and EPI (absolute and relative) weight were lower than in H group.

**Table 2:** Absolute (g) and Relative (g/100 g b.w) weight of the Retroperitoneal and Epididymal White Adipose Tissue in rats fed different diets during 8 weeks.

	Retroperitoneal		Epididymal	
	Absolute	Relative	Absolute	Relative
C	3.73 ± 0.29	0.87 ± 0.06	3.20 ± 0.40	1.23 ± 0.05
CPF	4.28 ± 0.34	1.02 ± 0.07	4.63 ± 0.33*	1.01 ± 0.07
H	5.34 ± 0.76	1.09 ± 0.17+	4.17 ± 0.43^+^	0.90 ± 0.08^+^
HPF	3.55 ± 0.43*	0.88 ± 0,09*	2.40 ± 0.14* °	0.66 ± 0.04*

Values are means ± SEM (n = 8).*p < 0.05 comparing C × CPF and H × HPF; ^+^p < 0.05 comparing C × H; °p < 0.05 comparing CPF × HPF. Control Group (**C**); Control + Fermented Product Group (**CPF**); Hypercholesterolemic Diet Group (**H**); and Hypercholesterolemic + Fermented Product Group (**HPF**).

**Figure 1:**
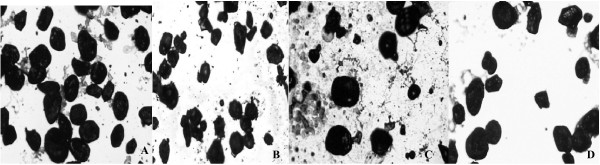
Circumference (μm) of the adipocyte from Retroperitoneal (RET) White Adipose Tissue in rats fed different diets during 8 weeks. Values are means ± SEM (n = 8). A – Control Group (C): circumference = 127.35 ± 2.49 μm. B – Control + Fermented Product Group (CPF): circumference = 118.06 ± 1.75* μm. C – Hypercholesterolemic Diet Group (H): circumference = 138.59 ± 2.37^+ ^μm. D – Hypercholesterolemic + Fermented Product Group (HPF): circumference = 113.49 ± 2.03* μm. *p < 0.05 comparing C × CPF and H × HPF; ^+^p < 0.05 comparing C × H.

**Figure 2:**
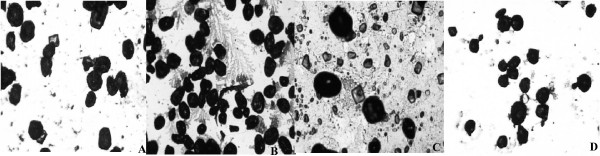
Circumference (μm) of the adipocyte from Epididymal (EPI) White Adipose Tissue in rats fed different diets during 8 weeks. Values are means ± SEM (n = 8). A – Control Group (C): circumference = 128.25 ± 2.05 μm. B – Control + Fermented Product Group (CPF): circumference = 110.13 ± 1.42* μm. C – Hypercholesterolemic Diet Group (H): circumference = 122.69 ± 1.73 μm. D – Hypercholesterolemic + Fermented Product Group (HPF): circumference = 110.12 ± 1.42* μm. *p < 0.05 comparing C × CPF and H × HPF.

A significant reduction was observed in RET and EPI adipocyte circumference when CPF and HPF were compared with C and H groups (Table 3 and Figure 1 and 2).

**Table 3:** Circumference (μm) of the adipocyte from Retroperitoneal (RET) and Epididymal (EPI) White Adipose Tissue in rats fed different diets during 8 weeks.

	Circumference	
Group	RET	EPI
C	127.35 ± 2.49	128.25 ± 2.05
CPF	118.06 ± 1.75*	110.13 ± 1.42*
H	138.59 ± 2.37^+^	122.69 ± 1.73
HPF	113.49 ± 2.03*	100.12 ± 1.42*

Values are means ± SEM (n = 8).*p < 0.05 comparing C × CPF and H × HPF; ^+^p < 0.05 comparing C × H. Control Group (**C**); Control + Fermented Product Group (**CPF**); Hypercholesterolemic Diet Group (**H**); and Hypercholesterolemic + Fermented Product Group (**HPF**).

### Measurements of the lipogenesis and lipolysis rate

The fermented soy product treatments significantly decreased lipogenesis rate in RET, EPI and Liver when H and HPF were compared. In CPF group, it was observed that RET and EPI lipogenesis rates were decreased when compared with C group, however, no significant differences were observed in liver lipogenesis rate (Table 4).

**Table 4:** In vivo lipogenesis rate (μmol of 3H2O incorporated into lipid per gram of tissue per hour) in RET, EPI and liver in rats fed different diets during 8 weeks.

Groups	RET	EPI	Liver
C	1,34 ± 0,29	0,59 ± 0,11	0,50 ± 0,10
CPF	0,29 ± 0,03*	0,14 ± 0,01*	0,61 ± 0,04
H	1,26 ± 0,11	1,30 ± 0,20^+^	2,94 ± 0,37^+^
HPF	0,52 ± 0,03*	0,03 ± 0,05* °	0,52 ± 0,03*

Values are means ± SEM (n = 8).*p < 0.05 comparing C × CPF and H × HPF; ^+^p < 0.05 comparing C × H; °p < 0.05 comparing CPF × HPF. Control Group (**C**); Control + Fermented Product Group (**CPF**); Hypercholesterolemic Diet Group (**H**); and Hypercholesterolemic + Fermented Product Group (**HPF**).

We observed a significant increase in lipolysis rate (RET and EPI) in the HPF group when compared with H. When C and CPF groups were compared it was observed an increase only in RET lipolysis rate (Table 5).

**Table 5:** In vitro lipolysis rate (μmol of glycerol released/h.100 mg) in RET and EPI in rats fed different diets during 8 weeks.

Groups	RET	EPI
C	0,34 ± 0,02	0,36 ± 0,01
CPF	0,51 ± 0,14*	0,29 ± 0,04
H	0,37 ± 0,02	0,39 ± 0,41
HPF	0,60 ± 0,003*	0,61 ± 0,04* °

Values are means ± SEM (n = 8).*p < 0.05 comparing C × CPF and H × HPF; ^+^p < 0.05 comparing C × H; °p < 0.05 comparing CPF × HPF. Control Group (**C**); Control + Fermented Product Group (**CPF**); Hypercholesterolemic Diet Group (**H**); and Hypercholesterolemic + Fermented Product Group (**HPF**).

### Plasma lipids

A significant TC and plasma TG is noted higher in comparing the H (108.67 ± 5.22; 177.83 ± 15.78) to the C Group (62.17 ± 3.07; 122.67 ± 15.08). Rats treated with hypercholesterolemic diet plus fermented product (HPF) presented a significant reduction of circulating cholesterol and triglyceride levels as compared to hypercholesterolemic diet group (Table 6).

**Table 6:** Lipid parameters (Plasma Triglycerides, Total Cholesterol and HDL-Cholesterol Concentrations – mg/dl) in rats fed different diets during 8 weeks.

Groups	Triglycerides	Cholesterol	HDL-cholesterol
C	122.67 ± 15.08	62.17 ± 3.07	18.66 ± 1.76
CPF	157.29 ± 16.36	69.28 ± 2.61	27.14 ± 1.71*
H	177.83 ± 15.78^+^	108.67 ± 5.22^+^	20.00 ± 1.26
HPF	94.83 ± 6.36*	91.40 ± 2.13* °	22.33 ± 1.02

Values are means ± SEM (n = 8).*p < 0.05 comparing C × CPF and H × HPF; ^+^p < 0.05 comparing C × H; °p < 0.05 comparing CPF × HPF. Control Group (**C**); Control + Fermented Product Group (**CPF**); Hypercholesterolemic Diet Group (**H**); and Hypercholesterolemic + Fermented Product Group (**HPF**).

Fermented soy product in HPF group tended to an increase (p = 0,08) in HDL-cholesterol concentration compared with H group. However, the difference was only significant when C group was compared with CPF group (Table 6).

## Discussion

In the present study, feeding rats with hypercholesterolemic diet resulted in a significant increase in food intake and body weight gain. On the other hand, previous reports did not show a difference significantly in food intake of rats fed chow diet and hypercholesterolemic diet [24-27]. Probably this could be accounted by the difference of the period of treatment and the amount of cholesterol present in the diet, since in our study the period of treatment was longer and the concentration of cholesterol was lower than made by the others [24-27]. In spite of that, the diet used in the present study was able to promote hypercholesterolemia and increase plasma triglycerides around 45% in rats, without modify HDL-cholesterol concentration.

This study demonstrated that a cholesterol-rich diet can significantly increase body weight gain, probably because of the increased food intake, which can cause dyslipidemia and obesity because of the additional cholesterol in its properties [28]. Several animal and human studies have confirmed the hypercholesterolemic properties of a cholesterol diet, which include increasing TC, TG, and alterations in the lipoprotein pattern, however, the mechanisms of which remain under study [28-30].

In rats, the mechanisms that prevent dyslipidemia are due to the reduction of the feedback inhibition of cholesterol biosynthesis and increased bile acid excretion, leading to a minor elevation of serum cholesterol after a cholesterol-enriched diet. [31]. However, cholic acid addition to the diet caused an increase in plasma total cholesterol levels, since cholic acid promotes cholesterol absorption [28] which was clearly shown in our study.

Fermented soy product administration resulted in a decrease in food intake and body weight gain in hypercholesterolemics rats, whereby the obtained values were similar in the control group that received or not the fermented soy product. Besides was observed a weight reduction of the white adipose tissue in hypercholesterolemic rats treated with fermented soy product. Previous study have found that the relative EPI weight in rats fed the cholesterol-enriched diet and a chow diet plus fermented soy product, tended to be lower than those in rats fed the chow diet or a cholesterol-enriched diet [24].

Our results showed a significant reduction in the circumference of EPI and RET depots when the ferment soy product enriched with *Enterococcus faecium *and *Lactobacillus Jugurti *was used, probably due to the significantly decrease in lipogenesis rate and increase in lipolysis rate in the RET and EPI, which were clearly shown in this study (H × HPF and C × CPF). Previous work has indicated that soy protein alters hormone balance and in turn accelerates lipid metabolism [21] and suppress hepatic lipogenic enzyme gene expression in Wistar fatty rats [32]. Our results suggested that a small amount of soy protein enriched with *Enterococcus faecium *and *Lactobacillus Jugurti *could have the same effect of the previously reported.

Some studies have reported a hypocholesterolemic effect with soy [19,33,34], whereas other studies have failed to find this effect [35]. In our study, the treatment with fermented soy product produced a significant lower in plasma cholesterol and triglyceride concentrations in the rats fed a hypercholesterolemic diet. Our results are in agreement with those reported by Rossi et al. [25] and Manzoni et al. [24], however in the last study was used the fermented soy product supplemented with isoflavones. Our results suggest that beyond isoflavone the *Enterococcus faecium *and *Lactobacillus Jugurti *can assist in the hypocholesterolemic effect. In most of the studies that reported positive results, was more effective in hypercholesterolemic subjects than in normocholesterolemic subjects [36,37].

There are multiple mechanisms by which soy protein could modulate plasma lipid and lipoprotein concentrations [37]. The interaction of protein with isoflavones and other non-protein components may contribute to the cholesterol-lowering effect of soy protein, since the isoflavones are structurally similar to estrogen and bind to the estrogen receptor [20,38]. In previous studies [39-42], reported that isoflavones have no effect when given separately.

Others soy bioactive components such as amino acids, minerals and the phytin acid could be acting actively in the decrease of the cholesterol and triglycerides. Same amino acids as the lysine increase the plasma cholesterol concentration [43], while others as e.g. arginine [44], have opposite action. This fact could partly be an explanation of hypocholesterolemic effects of the soy, as it contains good relation arginine/lysine [45]. The phytin acid present in soy has chelant property for certain minerals, such as for instance zinc. As the soy reduces the zinc absorption, it provides a low relation zinc/copper, which could reduce cholesterol. However, the concentration of these substances was not evaluated in the present study.

It is known that the soy presents some antinutritious factors such as trypsin and chymotrypsin inhibitors, mineral chelants and flatulence factor. Studies consider the possibility that trypsin inhibitors are capable to increase the cholecistochinin secretion that stimulates the liberation and subsequent bile secretion [42,43]. The soy possesses great fibre amount so this could be as well related to its hypocholesterolemic effects [44]. In addition, as was stated by Kritchevsky [45], animals fed soy protein excrete more neutral and acidic steroids, and have increased activity of hepatic HMG CoA reductase and cholesterol 7alpha-hydroxylase. In fact, Aoyama et al. [14] showed that rats fed soy protein had an increase in fecal-fat excretion.

The addition of the Enterococcus faecium to soy also could contribute to the decrease in cholesterol concentration in the group treated with hypercholesterolemic diet, this effect was possibly due to the ability of the *E. faecium *to reduce in 54% the cholesterol added to the adequate culture medium [26], it has been proposed that the possible hypocholesterolemic effect of *E. faecium *involves inhibition of exogenous cholesterol absorption from the diet [24].

Rossi et al. [25], reported that administration of fermented soy product enriched with *Enterococcus faecium *and *Lactobacillus Jugurti *10 ml/day, for 15 days lowered in plasma cholesterol in 18,4%, however this difference disappeared at the 30th day. As was stated by St-Onge et al. [46], the fermented product containing several types of bacteria, with indigestible carbohydrates and that produce short-chain fatty acid that will alter cholesterol synthesis. Also, the intestinal bacteria can bind bile acids to cholesterol, resulting in the excretion of bile acid-cholesterol complexes in the feces. Decreased bile acid recycling through the enterohepatic circulation would result in cholesterol uptake from the circulation into the liver for again synthesis of bile acids.

The fermented soy product use promoted a significant increase in HDL-cholesterol concentration, this is an important factor that determining the reduction in the risk of cardiovascular diseases. Similar results were obtained by Fukushima and Nakano [47] and Rossi [25], who showed that rats fed with high cholesterol diet, had reduction in the total cholesterol and an increase in HDL fraction with a daily intake of a probiotic product containing species of *Lactobacillus*, *Streptococcus*, and yeast.

The intake of 200 ml/day of the fermented soy milk, produced with *Enterococcus Faecium *and *Lactobacillus Jugurti*, for 6 weeks led to a 10% increase in HDL-cholesterol level in normocholesterolemic middle-age men [48]. Agerholm-Larsen et al. [49], reported that the consumption of yogurt fermented with different probiotics had variable effects on LDL cholesterol in obese subjects. Previous studies that used probiotic mixture containing *Lactobacillus acidophilus, Lactobacillus casei*, and *Bifidobacterium bifidum*, but found no significant effect on body weight, fat deposition, plasma cholesterol levels [50], suggesting that hypolipidemic effect is dependent on the type of probiotics.

In addition, the low serum cholesterol concentrations observed in rats treated with HPF diet as compared to H diets; might be due to the reduction of lipogenesis. Thus, the reduced biosynthesis of fatty acids in turn will reduce the production of VLDL particles, thus limiting the formation of LDL particles and resulting in low serum triglycerides and cholesterol concentrations [51].

Further studies are needed to clarify whether this effect is due to the soy protein or to the *Enterococcus faecium *and *Lactobacillus Jugurti and *whether other types of probiotic mixtures containing different bacterial strains, other than used in the present study, have effects on lipid metabolism deserves further investigation.

## Conclusion

The present study showed that 1 ml/day/rat of fermented soy product enriched with Enterococcus faecium and Lactobacillus Jugurti induces an improvement in lipid profile and reduction in visceral and central adiposity.

## Methods

### Animals

Thirty-two adults male Wistar rats, weighing 210 ± 10 g were housed in individual cages. The animals were kept at an environmental temperature of 23 ± 2°C in a 12 h light/dark controlled room. All animal procedures were performed according to principles in the Guide for the Care and Use of Laboratory Animals [52].

### Diets

Before beginning the experiment, the animals were submitted during five days to an adaptive period of flavour conditioning: during two days the animals received the sweet solution; afterwards they received during two days the sweet and acid solution, and finally the placebo product. The animals were randomly divided into four groups (n = 8) as follows: Control Group (C); Control + Fermented Product Group (CPF); Hypercholesterolemic diet group (H); and Hypercholesterolemic + Fermented Product Group (HPF). The control groups were fed with a chow diet (Nuvilab^®^). The animals of the H and HPF groups were fed with a chow diet enriched in cholesterol 0,15% (w/w), of Sigma C 8503 cholesterol. The cholesterol was diluted in ethyl ether P.A and stabilized with 10 ppm of butylated hydroxytoluene-BHT. The HPF and CPF groups received an intragastric administration of 1 ml of fermented product daily, during the 8 weeks of the study. Body weight gain and total food intake was measured.

### Preparation of the fermented product

The fermented soy product was prepared according to the methodology described by Rossi et al. (1999), and was added in 1.5% (v/v) of the *Enterococcus faecium *CRL 183 culture and 1.5% (v/v) of *Lactobacillus Jugurti *416 culture. Quantification of the viable cells in the finished fermented product was performed using the specific M 17-agar and MRS-agar media of culture. The colonies formed were counted and their morphological characteristics were recorded [26].

### Experimental Procedure

After 8 weeks of treatment the animals were killed by decapitation. Trunk blood was collected in a heparinized tube and was centrifuged for 15 min at 2500 rev./min. Retroperitoneal (RET) and Epididymal (EPI) white adipose tissues were immediately removed and weighed.

### Adipocyte size

A fragment (100 mg) of RET and EPI was fixed in 0.2 M collidine buffer pH 7.4, containing 2% of osmium tetroxide at 37°C. After 24 hours, they were washed with warmed saline as described by Hirsch & Gallian (1968). The adipocyte circumference was measured using image analysis software (Image Pro Plus) and expressed as μm.

### Plasma lipids

Plasma was used to measure triglycerides (TG), total cholesterol (TC) and HDL-cholesterol. For these measurements we used commercial Kits from Labtest Diagnostic S.A.

### Measurements of the lipogenesis rate

All animals were killed by decapitation 1 h after an intraperitoneal injection of 3 mCi 3H2O in a volume of 0.3 ml for the determination of the in vivo lipogenesis rate. The "de novo" lipogenic (fatty acid synthesis) rate was determined by the incorporation of 3H2O into saponified lipids according to the method of Robinson & Williamson [53]. Tissues samples were digested in 3 ml of 30% KOH and 3 ml of ethanol for at least 2 h at 70°C in capped tubes for the lipids to saponify. After cooling, 2 ml of 12 N H2SO4 was added, and lipid was extracted three times with 10 ml of petroleum ether [54]. The combined petroleum ether plus extracts was washed with 2 ml of distilled water and evaporated to dryness. The extracted fatty acids were dissolved in 5 ml of scintillation liquid, and radioactivity of 20 μl serum samples was counted for the determination of specific activity. The rate of lipogenesis was calculated as micromoles of 3H2O incorporated into lipids per gram per hour. The lipid content of the tissue was determined by the gravimetric method [55].

### Determination of lipolysis rate "in vitro"

Tissue fragments (about 100 mg) of RET and EPI were used for "in vitro" determination of glycerol release, an index of lipolytic rate [56]. The samples were minced into small fragments and incubated for 1 h at 37°C under continuous shaking in Ca2+-free Krebs-Henseleit solution containing 2% (w/v) bovine albumin (fraction V-essentially fatty acid-free), pH 7.4. Lipolysis was interrupted by placing the vials on ice. The tissue fragments were then removed and the glycerol content in the medium was determined enzymatically by the method of Eggstein and Kreutz [57]. The results were expressed as μmol of glycerol released/h.100 mg of tissue.

### Statistical Analysis

Results are expressed as means ± standard error of the means. Data were analyzed using two-way ANOVA to test the effects of diets, fermented soy product and their interactions, following by Tukey-Kramer multiple comparison test. Significance was accepted at the p < 0.05 level.

## Abbreviations

BHT: Butylated hydroxytoluene; C: Control Group; CPF: Control + Fermented Product Group; E.: Enterococcus; EPI: Epididymal adipose tissue; H: Hypercholesterolemic diet group; HDL: High Density Lipoprotein; L.: Lactobacillus; LDL: Low Density Lipoprotein; PF: Fermented Product Group; RET: Retroperitoneal adipose tissue; TC: Total cholesterol; TG: Triglycerides; VLDL: Very Low-Density Lipoprotein

## Competing interests

The authors declare that they have no competing interests.

## Authors' contributions

All authors have read and approved the final manuscript.

NCC made contributions to the conception and design of the study, generation, collection, assembly, analysis and interpretation of data and drafting the manuscript. ARD made contributions to the conception and design of the study, generation, collection, assembly, analysis and interpretation of data and drafting the manuscript. EAR made contributions to the conception and design of the study, generation, collection, assembly, analysis and interpretation of data and drafting the manuscript. RFLG made contributions to the collection, assembly, analysis of data and revision of the manuscript.

FPV made contributions to the collection, assembly, analysis of data and revision of the manuscript. MSJM made contributions to the collection, assembly, analysis of data and revision of the manuscript. IZC performed the design of the study and interpretation of data. PLS performed the design of the study and interpretation of data. RCV performed the design of the study and interpretation of data. CMON performed the design of the study and interpretation of data.
